# Differences in Outpatient Health Care Utilization 12 Months after COVID-19 Infection by Race/Ethnicity and Community Social Vulnerability

**DOI:** 10.3390/ijerph19063481

**Published:** 2022-03-15

**Authors:** Sarah E. Roth, Diana J. Govier, Katherine Marsi, Hannah Cohen-Cline

**Affiliations:** Center for Outcomes Research and Education (CORE), Providence, 5211 NE Glisan Street, Portland, OR 97213, USA; diana.govier@providence.org (D.J.G.); katherine.marsi@providence.org (K.M.); hannah.cohen-cline@providence.org (H.C.-C.)

**Keywords:** ambulatory care, disparity, health care utilization, coronavirus

## Abstract

Ensuring access to high-quality outpatient care is an important strategy to improve COVID-19 outcomes, reduce social inequities, and prevent potentially expensive complications of disease. This study assesses the equity of health care response to COVID-19 by examining outpatient care utilization by factors at the individual and community levels in the 12 months prior to and following COVID-19 diagnosis. Employing a retrospective, observational cohort design, we analyzed electronic health record data from a sample of 11,326 adults diagnosed with COVID-19 between March and July 2020. We used two-part models to estimate changes in use of primary and specialty care by race/ethnicity and community social vulnerability in the year before and after COVID-19 diagnosis. Our findings showed that while overall probability and counts of primary and specialty care visits increased following a positive COVID-19 diagnosis, disparities in care utilization by race/ethnicity and living in a socially vulnerable community persisted in the year that followed. These findings reiterate the need for strategic approaches to improve access to and utilization of care among those diagnosed with COVID-19, especially for individuals who are traditionally undeserved by the health system. Our findings also highlight the importance of systematic approaches for addressing social inequity in health care.

## 1. Introduction

Since January 2020, there have been over 79,000,000 cases and over 960,000 deaths related to coronavirus disease (COVID-19) in the United States (U.S.) [1]. The COVID-19 pandemic has had an immense effect on daily lives across the globe, including unprecedented impacts on health care systems and health care utilization [2,3]. As critical care units struggled to expand capacity as waves of infections surged, public concern around safety coupled with the deprioritization of nonurgent health care led to a substantial decrease in overall patient volumes. Research conducted in the early months of the pandemic found mixed patterns of care utilization that differed for patients living in areas of lower income and predominantly comprising racial/ethnic minorities, with disparities increasing for some and closing for other traditionally marginalized groups [4,5]. Given the ongoing scale, scope, and duration of the pandemic, it is essential to expand our understanding around equity of health care access and utilization, particularly for those diagnosed with COVID-19.

A mounting body of evidence points to increased use of health care services following COVID-19 diagnosis as many as nine months or more after disease onset [6,7]. While evidence from early in the pandemic found disproportionate rates of infection, disease severity, hospitalization, and death among racial/ethnic minorities as well as among populations with higher levels of community social vulnerability [8,9,10,11,12], studies that have examined care utilization following COVID-19 diagnosis have not assessed the ways in which social equity may have permeated patterns of care use for those with COVID-19 [6,7]. In addition, there is increased concern that the long-term symptoms and sequalae associated with COVID-19 will disproportionately impact patient populations along these same dimensions [13]. As such, it is essential to characterize equity of care use following COVID-19 diagnosis to improve population health management of COVID-19 moving forward.

The type, extent, and manifestation of COVID-19 symptoms are broad and highly variable, potentially impacting multiple organ systems [14,15,16]. Moreover, the effects of COVID-19 can be long ranging. As many as a third of those with COVID-19 report at least one persistent symptom several months after their initial diagnosis [14,17]. Even mild COVID-19 may be associated with long-term symptoms, most commonly cough, low-grade fever, and fatigue, all of which may relapse and remit [18]. Given the overall high rates of infection in the U.S. along with the high rates of extended symptoms, understanding the health care needs of those recovering from COVID-19 is essential to support clinicians and health care systems in provision of care [6].

The connection between systemic and structural inequities and the distribution of illness is often brought to the forefront during public health emergencies, and the COVID-19 pandemic has been no exception. COVID-19 has exacerbated existing health disparities in the U.S. context. Notably, racial and ethnic minority groups and socially vulnerable communities, such as those characterized by poverty, limited English proficiency, and crowded housing [8,9], have been disproportionately impacted by COVID-19 [10,11,12]. Furthermore, there is heightened concern that these same marginalized groups will bear an inequitable burden of post-acute COVID-19 syndrome [13].

Health care utilization following COVID-19 diagnosis can provide key insights into the equity of health care access during this period. Access to high-quality outpatient care, comprising primary care and specialty care, is essential to reducing preventable complications of conditions and thus essential to decreasing rates of hospitalization and death [19,20]. Andersen’s Behavioral Model of Health Services points to predisposing (e.g., demographics and social structure) and enabling characteristics (e.g., community attributes) as key factors shaping the equity of care access and utilization [21]. Ensuring access to high-quality outpatient care is an especially important strategy following cases of COVID-19 to improve outcomes, reduce social inequities, and prevent potentially expensive complications of disease [18]. Given that health care access can be broadly defined as “the timely use or personal health services to achieve the best outcomes”, we relied on outpatient care utilization metrics as our proxy measure for access in this study [22].

To date, few studies have examined longer-range patterns in outpatient health care utilization following COVID-19 diagnosis. Preliminary studies have shown increased rates of outpatient and primary care utilization in the months following a positive COVID-19 test [6,7]. For instance, a study of patients in a health system in Georgia found that overall outpatient utilization rates were high following cases of mild or moderate COVID-19, with nearly 70% of the sample having at least one visit during the six-month follow up period [6]. However, these studies do not account for the ways in which social inequity may permeate patterns of post-COVID-19 health care use.

Using electronic health record data from a large health system in western U.S., this study aimed to address gaps in understanding around the equity of health care response to COVID-19 by examining how or if disparities in outpatient care utilization by race/ethnicity and community social vulnerability changed in the 12 months following COVID-19 diagnosis. The findings of the research can be used to support the development of more effective disease management strategies for those most impacted by COVID-19.

## 2. Methods

### 2.1. Study Data and Population

This study used patient demographic, health characteristics, and health care utilization data from the Providence health system electronic health record (EHR). Providence operates nonprofit health care systems across Oregon, Washington, California, Montana, New Mexico, Texas, and Alaska [23]. Using these data, we employed a retrospective, observational study design to examine the associations between race/ethnicity, community social vulnerability, and health care utilization among patients diagnosed with COVID-19 at a Providence site between March and July 2020, comparing primary and specialty care utilization one year before and after COVID-19 diagnosis. Individuals were included in the study if they were at least 18 years of age at the time of their COVID-19 diagnosis, did not die during the year after their COVID-19 diagnosis, and resided in a state where Providence operates other than Texas (at the time of this study, Texas’ EHR was not fully integrated with the larger Providence EHR); this resulted in 12,398 identified individuals. Individuals were then excluded from the study if they were missing community social vulnerability (*n* = 31) and race/ethnicity (*n* = 1072) data. With these criteria, the final study sample comprised 11,326 individuals.

### 2.2. Variables

#### 2.2.1. Dependent Variables

Our dependent variables were primary care utilization, defined as a visit with a primary care provider (PCP), and specialty care utilization, defined as an outpatient visit with a non-PCP (e.g., cardiologist or nephrologist). For each outcome, we looked at both binary (yes/no to a visit) and count (number of visits) variables in the year before and the year after COVID-19 diagnosis. Visit count and type were identified via the EHR. Because we were interested in long-term health care utilization associated with COVID-19 diagnosis, we censored visits that took place during the first 30 days after COVID-19 diagnosis as these may have been associated with acute disease care [6]. From these, we created binary variables for whether an individual had a visit before and after their COVID-19 diagnosis for each visit type, again censoring visits that took place during the first 30 days after COVID-19 diagnosis.

To test the sensitivity of our outcome variables to censoring of the first 30 days after COVID-19 diagnosis, we also created binary and count variables for PCP and specialty care utilization in which we did not censor the first 30 days after COVID-19 diagnosis.

#### 2.2.2. Main Independent Variables

Our main independent variables included a binary variable for the post-COVID-19 diagnosis period, binary variables for four dimensions of community social vulnerability, and a mutually exclusive categorical variable for race/ethnicity. The post-COVID-19 diagnosis period variable identified whether the observation occurred before or after an individual’s COVID-19 diagnosis.

Community social vulnerability was determined using the Social Vulnerability Index (SVI) from the Centers for Disease Control and Prevention, which signifies a community’s social vulnerability due to factors such as poverty, lack of access to transportation, and crowded housing [24,25]. The SVI ranks each tract on 15 social factors, which are grouped into four dimensions of vulnerability: (1) socioeconomic status, (2) household composition and disability, (3) minority status and language, and (4) housing type and transportation [26,27]. The SVI determines the ranking of each census tract via a percentile ranking of the proportion of tracts that are equal to or lower than the tract of interest. For example, a tract ranking of 0.90 indicates that the tract in question is more vulnerable than 90 percent of tracts in that state along the social factor in question [28,29]. From this, we created binary variables for each of the four SVI dimensions. These variables were equal to 1 if an individual resided in a tract that was more vulnerable in that SVI dimension than 90 percent or more of the tracts in their state, and 0 otherwise.

Data on study participants’ race and ethnicity was used to create a mutually exclusive categorical race/ethnicity variable comprising seven categories: non-Hispanic White, non-Hispanic Black, non-Hispanic Asian, non-Hispanic American Indian/Alaskan Native (AIAN), non-Hispanic Native Hawaiian/Pacific Islander (NHPI), non-Hispanic Other race, and Hispanic/Latino. If a study participant was classified as Hispanic/Latino and any other race, they were categorized as Hispanic/Latino, otherwise they were categorized based on the most recent racial information available in the EHR.

#### 2.2.3. Covariates

Covariates included age, calculated as the study participant’s age at the date of their COVID-19 diagnosis and grouped into seven categories (<20, 20–34, 35–44, 45–54, 55–64, 65–74, and ≥75); sex (male, female, and other/unknown); primary language spoken (English, Spanish, other, and unknown); payor type (commercial, Medicare, Medicaid, other government, other miscellaneous, and unknown); state of residence (California, Oregon, Washington, and other); whether a study participant was diagnosed with COVID-19 in an inpatient setting (yes/no); and five binary variables indicating whether a study participant had chronic health conditions of diabetes, hypertension, coronary artery disease, chronic kidney disease, and congestive heart failure. These conditions were the five most common conditions among the study sample that were flagged by Providence as potentially contributing to worse COVID-19 disease and outcomes.

### 2.3. Statistical Approach

#### 2.3.1. Descriptive Analysis

To examine characteristics of our study sample, we computed cell sizes and proportions for categorical variables. We descriptively examined our health care utilization outcomes by computing means, standard deviations, minimums, medians, and maximums for the entire study period and stratified by the pre- and post-COVID-19 diagnosis period.

#### 2.3.2. Regression Analysis

To examine changes from the pre- to post-COVID-19 diagnosis period in health care utilization by SVI dimension and race/ethnicity, we created interaction terms for the binary post-COVID-19 diagnosis period and each binary SVI dimension variable as well as interaction terms for the binary post-COVID-19 diagnosis period and the categorical race/ethnicity variable.

We employed two-part models, which provide a unified approach to account for mass points at zero, to explore changes in health care utilization from the pre- to post-COVID-19 diagnosis period by community social vulnerability dimension and race/ethnicity [30]. In the first part of the model, binary choice models were fit using logistic regression, which modeled the probability of observing a positive (versus zero) count of each utilization outcome. Conditional on a positive visit count, we fit truncated Poisson regression models to examine visit counts for each utilization outcome. For each utilization outcome, we calculated average marginal effects as predicted probabilities for part 1 and as visit counts for part 2 of our two-part models. Our main models included the main independent variables described above and covariates for age group and sex. In ancillary models, we included the main independent variables and all covariates described above.

To test the sensitivity of our results to censoring of the first 30 days after COVID-19 diagnosis, we ran the same two-part models described above but included binary and count outcomes for PCP and specialty care utilization that did not include censoring of the first 30 days after COVID-19 diagnosis.

To correct for heteroskedasticity, we estimated robust standard errors for all models [31]. All analyses were performed using Stata version 14.2. This study was reviewed and approved by the Providence Institutional Review Board.

## 3. Results

### 3.1. Study Population Characteristics

More than half of individuals in the study population were categorized as residing in a vulnerable area along at least one dimension of community social vulnerability, with 19.6% categorized as residing in a vulnerable area based on socioeconomic status, 22.1% based on household composition, 23.5% based on minority status/language, and 41.6% based on housing type/transportation (Table 1). Individuals were primarily of Hispanic/Latino (41.6%) and non-Hispanic White (39.5%) race/ethnicity, while non-Hispanic Black, Asian, NHPI, AIAN, and other race/ethnicity individuals each comprised 5.5% or less of the study population. The majority of individuals were aged 20 to 64; were female; had English as their primary language; and resided in California, Washington, or Oregon. Approximately one-quarter of individuals were diagnosed with COVID-19 in an inpatient setting. In addition, one-quarter had hypertension, while a smaller proportion had diabetes (14.8%), coronary artery disease (10.4%), chronic kidney failure (9.2%), and congestive heart failure (4.8%).

### 3.2. PCP and Specialty Visit Utilization

Overall, less than one-third of individuals in the study population had at least one PCP visit during the study period, while almost half had at least one specialty care visit during the study period. The average number of specialty care visits utilized was greater than the average number of PCP visits utilized during the study period (2.69 specialty care visits vs. 1.31 PCP visits per person) (Table 2). Compared with the period before COVID-19 diagnosis, the proportion of individuals who utilized PCP and specialty care both increased after COVID-19 diagnosis, as did the average number of PCP and specialty care visits utilized. Visit counts were highly skewed, with the median number of visits for both PCP and specialty care equal to zero over the entire study period and before and after COVID-19 diagnosis.

### 3.3. Community Social Vulnerability and PCP and Specialty Visit Utilization

In our main models that adjusted for age and sex, we observed significant differences in the likelihood of PCP and specialty care use by community social vulnerability status prior to COVID-19 diagnosis (Table 3 and Table 4). For example, individuals residing in areas categorized as vulnerable based on minority status/language and housing type/transportation were less likely to utilize primary and specialty care prior to their COVID-19 diagnosis, while individuals residing in areas categorized as vulnerable along the household composition dimension were less likely to utilize specialty care before their COVID-19 diagnosis. On the other hand, for both PCP and specialty care visits, conditional on having at least one PCP or specialty care visit, dimensions of community social vulnerability were not significantly associated with the number of PCP or specialty care visits individuals used before their COVID-19 diagnosis.

Interaction terms for community social vulnerability and post-COVID-19 diagnosis were insignificant, indicating that differences in primary and specialty care use by community social vulnerability did not change significantly after COVID-19 diagnosis. SVI was still associated with the likelihood of having a PCP and specialty care visit but not with the number of visits utilized. The results of our sensitivity analysis in which we did not censor the first 30 days after COVID-19 diagnosis were qualitatively similar to our main results (data not shown). 

In ancillary models that adjusted for the additional covariates of COVID-19 diagnosis in an inpatient setting primary language spoken, state of residence, payor, and chronic conditions, negative relationships between dimensions of community social vulnerability and PCP and specialty care use were largely attenuated. As in our main models, there were no significant differences before or after COVID-19 diagnosis in the number of PCP or specialty care visits by community social vulnerability status conditional on having at least one PCP or specialty care visit.

### 3.4. Race/Ethnicity and PCP and Specialty Visit Utilization

For both PCP and specialty care, race/ethnicity was significantly associated with the likelihood of care use in our main models that adjusted for age group and sex (Table 3 and Table 4). For PCP visits, prior to COVID-19 diagnosis, individuals of non-Hispanic NHPI, non-Hispanic AIAN, non-Hispanic other, and Hispanic/Latino race/ethnicity were less likely to have a visit than non-Hispanic White individuals.

Regarding the amount of care used, prior to COVID-19 diagnosis, non-Hispanic Black individuals with at least one PCP visit used a significantly greater number of PCP visits than non-Hispanic White individuals with at least one PCP visit. For all other racial/ethnic groups, there were no significant differences in the amount of PCP or specialty visits used conditional on having at least one PCP or specialty care visit.

The interaction terms for race/ethnicity were largely insignificant, with one exception. Among non-Hispanic Asian individuals, after COVID-19 diagnosis, their likelihood of using specialty care increased significantly. All other interaction terms for race/ethnicity and post-COVID-19 diagnosis, including interactions terms for number of visits used conditional on having at least one visit, were insignificant, indicating that these differences persisted after COVID-19 diagnosis. Results from our sensitivity analysis in which we did not censor the first 30 days after COVID-19 diagnosis were qualitatively similar to our main results (data not shown).

After adjusting for additional covariates in ancillary models, negative relationships between race/ethnicity and the likelihood of PCP and specialty visit use were largely attenuated, except among non-Hispanic Asian individuals. In contrast, positive but insignificant differences in the likelihood of PCP visit use prior to COVID-19 diagnosis for some racial/ethnic groups (non-Hispanic Black and non-Hispanic Asian) became significant after COVID-19 diagnosis. Conditional on having at least one PCP or specialty visit, associations between race/ethnicity and the number of PCP and specialty visits used did not materially differ between the main and ancillary models and were mostly insignificant both before and after COVID-19 diagnosis.

## 4. Discussion

Unprecedented disruptions in health care provision, coupled with a limited knowledge of the long-term sequalae of the COVID-19 virus, highlight the need to understand how access to and utilization of outpatient care have changed during this pivotal time. Likewise, disparities in COVID-19 infection rates, severity, and mortality by neighborhood characteristics and race/ethnicity, both in our research and previous studies, emphasize the imperative to examine access and utilization with a specific lens on equity.

Our findings show that while the overall probability and counts of primary and specialty care use increased following a positive COVID-19 diagnosis, disparities in utilization by race/ethnicity and living in a socially vulnerable community existed in the year before COVID-19 diagnosis and persisted in the year following diagnosis.

Both the likelihood and the amount of outpatient visits increased among the sample in the 12 months following COVID-19 diagnosis. Although the literature on this emergent topic remains scant, this finding aligns with previous work showing increases in outpatient care use in the months following COVID-19 infection. We found that specialty care use was more common than primary care use. This differs from prior work showing no change in specialty care use following mild cases of COVID-19 in the year following COVID-19 diagnosis [32]. There are at least two potential explanations for this. First, specialty care is often used to manage chronic conditions, and the rates of diabetes, coronary artery disease, and congestive heart failure in our sample were higher than national prevalence estimates for these conditions, suggesting a higher than average need for specialty care even before COVID-19 diagnosis [33,34,35,36]. Second, increased probability of specialty care utilization may also be related to the compounding effect of chronic conditions on the severity of COVID-19 illness and subsequent recovery [37]. Continued research is needed to understand the interplay between COVID-19, chronic disease, and health care utilization [38].

Importantly, our findings extend on previous work by looking at disparities in health care utilization following a positive COVID-19 diagnosis. These disparities also exist in the risk of COVID-19 infection itself; more than half of the sample in this study, all of whom tested positive for COVID-19 during the study period, lived in an area with relatively high community social vulnerability. Further, the majority identified as Hispanic/Latino, a disproportionate amount given that more than 70% of the overall Providence system patient population identifies as non-Hispanic White. In addition to disparities in the risk of disease, we saw disparities in access to and use of health care after infection.

With respect to community-level factors, measured by SVI, we observed significantly lower probability of primary care and specialty care utilization among individuals living in areas categorized as vulnerable along dimensions of minority/language and housing transportation type before their COVID-19 diagnosis even when accounting for patients’ race/ethnicity. A well-established body of evidence has demonstrated how neighborhood characteristics shaped by social stratification processes, such as residential segregation, community divestment, and the geographical concentration of poverty, determine differential access to and utilization of health care [39,40]. Environmental stressors, such as pollution, noise, and overcrowded and low-quality housing stock, can lead to illness and poor health [39]. Additionally, neighborhood characteristics help determine where an individual may receive health care, which in turn impacts the quality of care they receive [41]. Thus, the fact that disparities in care by community social vulnerability were observed before and after COVID-19 diagnosis is not overly surprising as the pandemic has continued to reveal and exacerbate numerous existing social inequalities.

Moreover, these health care disparities persisted after the COVID-19 diagnosis. These findings point to the need for outreach approaches tailored to individuals living in vulnerable communities. For example, mobile health clinics have been shown to be a cost-effective approach for improving health care access in underserved communities [42]. The nature of the pandemic has also given rise to virtual care delivery models, which, depending on how they are implemented, have the potential to help mitigate disparities in health care access [5,43]. Even so, it remains salient that inequities in our health systems are deeply entrenched and rooted in structural inequalities that require structural solutions to improve upon the status quo.

We also observed disparities in health care utilization by race/ethnicity even when controlling for community social vulnerability. Patients who identified as NHPI, AIAN, and Hispanic/Latino had lower probabilities of primary care and specialty care use in the year prior to COVID-19 diagnosis. These disparities in access did not change following COVID-19 infection. Like neighborhood characteristics, an individual’s race/ethnicity significantly shapes health care access in the U.S. [44,45]. Structural racism contributes to deep inequities in health care utilization and outcomes by race and ethnicity [46]. Not only does the experience of racism have a direct biological impact on health, increasing stress levels and worsening health outcomes [47], but the manifestation of structural racism in terms of income and employment opportunities also restricts access to health insurance, leading to delayed care and undertreatment [48]. At the same time, persistent racism experienced by racial and ethnic minority groups in health care settings also reduces care-seeking behavior and contributes to increased rates of morbidity and mortality [49]. One promising strategy that health care systems can employ to reduce disparities in access to care includes increasing the representativeness of the health care workforce, especially Black, Latinx, and Native American providers, which has been shown to improve access to care for racially and ethnically diverse populations regardless of income levels [50,51]. Future research and practice should work to identify and invest in successful approaches to recruit and retain a diverse population in the full spectrum of health care careers [52].

Patients who identified as Asian also had lower probabilities of specialty care utilization in the year prior to their COVID-19 diagnosis. However, we observed significant increases in the probability of specialty care use in the 12 months following a SAR-CoV-2 infection among Asian patients relative to White patients. Previous research has demonstrated that Asian individuals often fare better than White individuals on measures of health and health care access; however, these experiences vary widely when considering different Asian subgroups [53]. Future research should seek to further disaggregate race/ethnicity data to better understand how access and utilization may differ by subgroup.

While our main models adjusted only for age and sex, we constructed ancillary models in line with Andersen’s Behavioral Model of Health Services Use to explore the impacts of adjustments for other predisposing, enabling, and need-related health and health care variables, namely setting of diagnosis (inpatient vs. outpatient), payer type, language, chronic conditions, and state. Each of these variables is strongly related to race/ethnicity and community social vulnerability, and the relationships between social inequity and health care utilization reflects the nature of persistent structural racism in the U.S. [54]. Diagnosis setting can serve as a proxy for how severe COVID-19 was at the time of diagnosis, and racial/ethnic minorities and individuals with higher community social vulnerability were more likely to be diagnosed with more severe disease [8,9,10,11,12]. Likewise, marginalized groups have more limited access to high-quality health insurance, which in turn decreases access to health care [47]. Individuals who do not speak English, have low socioeconomic status, and identify as racial and/or ethnic minorities face discrimination in health care settings and often do not receive linguistically or culturally appropriate care. Racial/ethnic minorities and low-income populations experience disproportionate rates of chronic disease [55,56]. Finally, we included state in the ancillary models to account for differences in health care policies [57].

These are therefore our ancillary, and not main, models because the inclusion of the additional variables may “adjust away” the observed disparities in use of care by adjusting for different pathways throughout which social inequity and structural racism acts. For example, the decreased probabilities of primary care use among NHPI, AIAN, and Hispanic/Latino patients observed in the main models were no longer significant in the ancillary models; we saw similar results in the specialty care models, where differences by SVI and race/ethnicity became attenuated or nonsignificant in the ancillary models. In contrast, the increased probability of primary care use among Black and Asian patients became significant, suggesting that these health and health care variables play a different role in shaping access to care among these groups. The differences in significance observed in the main and ancillary models illustrate how disparities in care happen through a myriad of factors and highlight the need for multipronged systematic approaches to reduce impediments to care among vulnerable populations.

### Limitations

This study has several limitations. Our sample was limited to Providence patients in six states. As such, our findings may not be generalizable across the U.S. Even so, this study draws on data collected over a large geographical area and represents a diverse, multistate cohort of patients. In addition, our sample was drawn from patient populations tested for COVID-19 in the early days of the pandemic. As such, patients with milder cases of COVID-19 who were infected without knowing it or were not able to get tested may have been excluded from our sample. Furthermore, the use of EHR data limits the interpretation of our findings. For instance, we cannot account for utilization that took place outside of the Providence. Moreover, our models are limited to information contained within EHR data, which may not include all factors relevant to care utilization, such as household income and composition, health-seeking behaviors, and health values and beliefs. Even so, by including measures of census-tract-level socioeconomic status and re-source-related variables in our measure of community social vulnerability, we can account for some variability in these factors and their relationship to health care use. Our study also did not characterize the reason for visit, which makes it difficult to parse if care utilization was connected to COVID-19 infection or other health needs; however, we did include a 30-day censor period to more accurately capture health care utilization following the acute COVID-19 stage.

## 5. Conclusions

We found that disparities in primary and specialty care use by race/ethnicity and neighborhood characteristics persisted in the year following COVID-19 infection. Given disruptions in health care utilization globally, the results of this research may have implications that extend beyond the U.S. context. The findings of this study underscore how systemic barriers at the patient and community levels drive disparities in COVID-19 outcomes and may also drive disparities in health care access following COVID-19 diagnosis. As such, health care policymakers around the world should foreground the needs of their most vulnerable populations in working to treat cases of COVID-19 and mitigate its spread. The results of this study reiterate the need for strategic approaches to improve access to and utilization of care among those with COVID-19, especially for individuals who are traditionally marginalized in the health system, as well as the importance of systematic approaches for addressing social inequity in health care.

## Figures and Tables

**Table 1:** Characteristics of the study sample.

Characteristic	Frequency	Percentage
Total	11,326	100.0
Any community social vulnerability	6420	56.7
*Community Social Vulnerability Dimensions*		
Socioeconomic status	2223	19.6
Household composition	2502	22.1
Minority status/language	2656	23.5
Housing type/transportation	4716	41.6
*Race/Ethnicity*		
Non-Hispanic White	4471	39.5
Non-Hispanic Black	622	5.5
Non-Hispanic Asian	605	5.3
Non-Hispanic NHPI	225	2.0
Non-Hispanic AIAN	92	0.8
Non-Hispanic other	592	5.2
Hispanic/Latino	4719	41.7
*Age Group*		
<20	267	2.4
20–34	3191	28.2
35–44	1947	17.2
45–54	2021	17.8
55–64	1774	15.7
65–74	1098	9.7
≥75	1028	9.1
*Gender*		
Male	5411	47.8
Female	5915	52.2
*Preferred Language*		
English	8611	76.0
Spanish	2107	18.6
Other	587	5.2
Unknown	21	0.2
*State of Residence*		
California	4652	41.1
Oregon	1805	15.9
Washington	4565	40.3
Other	305	2.7
*Payer Type*		
Commercial	1939	17.1
Medicare	1595	14.1
Medicaid	2108	18.6
Other government	745	6.6
Other miscellaneous	202	1.8
Unknown	4737	41.8
*COVID-19 Diagnosis Encounter Setting*		
Inpatient	2715	24.0
Outpatient	8611	76.0
*Chronic Conditions*		
Chronic kidney disease	1046	9.2
Congestive heart failure	546	4.8
Coronary artery disease	1176	10.4
Diabetes	1673	14.8
Hypertension	2536	22.4

Notes: Abbreviations: NHPI, Native Hawaiian/Pacific Islander; AIAN, American Indian/Alaskan Native.

**Table 2:** PCP and specialty visit utilization overall and by pre- and post-COVID-19 diagnosis periods.

Utilization Outcome	Number with Any Visit (N/%)	Mean Number of Visits	Standard Deviation	Minimum Number of Visits	Median Number of Visits	Maximum Number of Visits
PCP visits, overall	3246 (28.66%)	1.31	3.09	0	0	64
Pre-COVID-19 period	2438 (21.53%)	0.61	1.61	0	0	25
Post-COVID-19 period	2667 (23.55%)	0.70	1.81	0	0	39
Specialty visits, overall	5167 (45.62%)	2.69	5.90	0	0	101
Pre-COVID-19 period	3607 (31.85%)	1.21	3.15	0	0	92
Post-COVID-19 period	3980 (35.14%)	1.48	3.62	0	0	61

Notes: Abbreviations: PCP, primary care provider.

**Table 3:** Probability and count of PCP visit utilization among individuals diagnosed with COVID-19.

	Model 1: Main Model		Model 2: Ancillary Model	
	Probability	Count	Probability	Count
Post-COVID-19 period	0.0070	0.20	0.0071	0.20
*Community Social Vulnerability Dimensions*				
Socioeconomic status	0.0045	−0.11	−0.0028	−0.12
Household composition	−0.015	0.034	−0.022 *	−0.0008
Minority status/language	−0.049 ***	0.25	0.023	0.28
Housing type/transportation	−0.059 ***	−0.17	−0.0046	−0.10
*Interactions between Community Social Vulnerability Dimensions and Post-COVID-19 Period*				
Socioeconomic status, post-COVID-19 period	0.0067	0.69	0.0065	0.67
Household composition, post-COVID-19 Period	0.0099	−0.19	0.0089	−0.19
Minority status/language, post-COVID-19 Period	0.0072	−0.54	0.0064	−0.51
Housing type/transportation, post-COVID-19 Period	−0.0031	−0.092	0.0026	−0.12
*Race/Ethnicity (Reference White)*				
Black	0.0051	0.81 *	0.046 **	0.68 *
Asian	0.013	−0.28	0.041 *	−0.18
NHPI	−0.066 **	−0.012	−0.0061	−0.23
AIAN	−0.111 ***	0.054	−0.048	0.066
Hispanic	−0.059 ***	0.22	0.017	0.22
Other	−0.068 ***	−0.15	−0.025	−0.15
*Interactions between Race/Ethnicity and Post-COVID-19 Period*				
Black, post-COVID-19 period	0.024	0.18	0.025	0.18
Asian, post-COVID-19 period	0.033	0.15	0.035	0.13
NHPI, post-COVID-19 period	0.010	0.82	0.010	0.80
AIAN, post-COVID-19 period	−0.025	−0.47	−0.025	−0.54
Hispanic, post-COVID-19 period	0.014	−0.0032	0.014	0.012
Other, post-COVID-19 period	0.035	0.15	0.034	0.15
*Age Group (Reference ≥ 75)*				
<20	−0.032	−1.76 ***	−0.25 ***	−1.17 ***
20–34	−0.049 ***	−1.50 ***	−0.24 ***	−0.94 ***
35–44	0.0022	−1.230 ***	−0.18 ***	−0.67 **
45–54	0.037 **	−1.26 ***	−0.13 ***	−0.66 ***
55–64	0.020	−0.67 ***	−0.12 ***	−0.27
65–74	0.054 ***	−0.14	−0.015	0.15
*Sex (Reference Female)*				
Male	−0.072 ***	−0.53 ***	−0.060 ***	−0.56 ***
Inpatient COVID-19 encounter	—	—	−0.087 ***	−0.096
*Primary Language (Reference English)*				
Spanish	—	—	−0.089 ***	0.12
Other	—	—	−0.084 ***	−0.28
Unknown	—	—	−0.14 **	−1.77 ***
*State (Reference California)*				
Oregon	—	—	0.093 ***	0.19
Washington	—	—	0.069 ***	0.23 *
Other	—	—	0.055 **	0.48
*Payer (Reference Commercial)*				
Medicare	—	—	−0.10 ***	0.22
Medicaid	—	—	−0.096 ***	0.51 *
Other government	—	—	−0.12 ***	−0.64 *
Miscellaneous	—	—	−0.081 ***	−0.074
Unknown	—	—	0.19 ***	0.070
*Chronic Conditions*				
Diabetes	—	—	0.013	0.43 **
Hypertension	—	—	0.056 ***	0.67 ***
Coronary artery disease	—	—	0.031 *	−0.017
Chronic kidney disease	—	—	0.027 *	0.18
Congestive heart failure	—	—	0.0049	0.036
Observations	22,652	5105	22,652	5105

Notes: Average marginal effects for factor levels computed based on discrete change from the base or reference level. Robust standard errors calculated using sandwich estimator. Model 1 includes variables for the post-COVID-19 diagnosis period (SVI themes, race/ethnicity, age group, and sex) and interactions between the post-COVID-19 diagnosis period and SVI themes and the post-COVID-19 period and race/ethnicity. Model 2 includes variables for the post-COVID-19 diagnosis period (SVI themes, race/ethnicity, age group, and sex), interactions between the post-COVID-19 diagnosis period and SVI themes and the post-COVID-19 period and race/ethnicity, and additional variables for inpatient COVID-19 diagnosis encounter setting (preferred language, state, payer, diabetes diagnosis, hypertension diagnosis, coronary artery disease diagnosis, chronic kidney disease diagnosis, and congestive heart failure diagnosis). * *p* < 0.05, ** *p* < 0.01, *** *p* < 0.001. Abbreviations: PCP, primary care provider; NHPI, Native Hawaiian/Pacific Islander; AIAN, American Indian/Alaskan Native.

**Table 4:** Probability and count of specialty visit utilization among individuals diagnosed with COVID-19.

	Model 1: Main Model		Model 2: Ancillary Model	
	Probability	Count	Probability	Count
Post-COVID-19 period	0.031 **	0.53 **	0.031 **	0.57 **
*Community Social Vulnerability Dimensions*				
Socioeconomic status	0.012	0.16	−0.0002	0.065
Household composition	0.027 *	0.30	0.014	0.17
Minority status/language	−0.083 ***	0.086	−0.0049	0.31
Housing type/transportation	−0.061 ***	−0.22	−0.0022	−0.0079
*Interactions between Community Social Vulnerability Dimensions and Post-COVID-19 Period*				
Socioeconomic status, post-COVID-19 period	−0.020	−0.0074	−0.020	0.016
Household composition, post-COVID-19 period	0.015	0.0020	0.015	0.0087
Minority status/language, post-COVID-19 period	0.015	0.022	0.013	0.039
Housing type/transportation, post-COVID-19 period	−0.023	−0.30	−0.023	−0.33
*Race/Ethnicity (Reference White)*				
Black	0.0037	0.13	0.035	0.048
Asian	−0.067 ***	−0.45	−0.044 *	−0.16
NHPI	−0.092 **	−0.11	−0.048	−0.23
AIAN	−0.012	1.59	0.049	1.40
Hispanic	−0.081 ***	0.033	−0.0034	0.35
Other	−0.010 ***	−0.71 *	−0.061 **	−0.52
*Interactions between Race/Ethnicity and Post-COVID-19 Period*				
Black, post-COVID-19 period	0.0073	0.58	0.0079	0.55
Asian, post-COVID-19 period	0.061 *	−0.22	0.060*	−0.29
NHPI, post-COVID-19 period	−0.0023	−0.20	−0.0018	−0.24
AIAN, post-COVID-19 period	0.0003	−1.32	0.0000	−1.26
Hispanic, post-COVID-19 period	0.0091	0.082	0.0099	0.039
Other, post-COVID-19 period	0.014	0.41	0.014	0.51
*Age Group (Reference ≥ 75)*				
<20	−0.069 **	−2.49 ***	−0.16 ***	−2.33 ***
20–34	−0.074 ***	−2.20 ***	−0.14 ***	−2.053 ***
35–44	−0.046 ***	−1.86 ***	−0.10 ***	−1.66 ***
45–54	−0.0097	−1.23 ***	−0.055 **	−1.065 **
55–64	0.0089	−0.66 *	−0.027	−0.56
65–74	0.042 **	−0.044	0.024	0.28
*Sex (Reference Female)*				
Male	−0.13 ***	−0.83 ***	−0.12 ***	−0.93 ***
Inpatient COVID-19 encounter	—	—	−0.061 ***	−0.47 *
*Primary Language (Reference English)*				
Spanish	—	—	−0.069 ***	−0.48 *
Other	—	—	−0.060 ***	−1.067 ***
Unknown	—	—	−0.28 ***	−3.33 ***
*State (Reference California)*				
Oregon	—	—	0.16 ***	0.60 **
Washington	—	—	0.11 ***	0.94 ***
Other	—	—	0.11 ***	0.55
*Payer (Reference Commercial)*				
Medicare	—	—	−0.086 ***	−0.72 **
Medicaid	—	—	−0.13 ***	0.39
Other government	—	—	−0.20 ***	−0.36
Miscellaneous	—	—	−0.071 **	−0.41
Unknown	—	—	0.11 ***	−0.13
*Chronic Conditions*				
Diabetes	—	—	−0.0017	0.069
Hypertension	—	—	0.055 ***	0.65 **
Coronary artery disease	—	—	0.077 ***	0.50
Chronic kidney disease	—	—	0.026	1.058 **
Congestive heart failure	—	—	0.034 *	0.38
Observations	22,652	7587	22,652	7587

Notes: Average marginal effects for factor levels computed based on discrete change from the base or reference level. Robust standard errors calculated using sandwich estimator. Model 1 includes variables for the post-COVID-19 diagnosis period (SVI themes, race/ethnicity, age group, and sex) and interactions between the post-COVID-19 diagnosis period and SVI themes and the post-COVID-19 period and race/ethnicity. Model 2 includes variables for the post-COVID-19 diagnosis period (SVI themes, race/ethnicity, age group, and sex), interactions between the post-COVID-19 diagnosis period and SVI themes and the post-COVID-19 diagnosis period and race/ethnicity, and additional variables for inpatient COVID-19 diagnosis encounter setting (preferred language, state, payer, diabetes diagnosis, hypertension diagnosis, coronary artery disease diagnosis, chronic kidney disease diagnosis, and congestive heart failure diagnosis). * *p* < 0.05, ** *p* < 0.01, *** *p* < 0.001. Abbreviations: NHPI, Native Hawaiian/Pacific Islander; AIAN, American Indian/Alaskan Native.

## Data Availability

The data used in this study was derived from Providence electronic medical records and included individual-level identifiers and protected health information. This is a closed database that is not available for public use.
